# Draft Genome Sequence of a Sulfur-Oxidizing Autotroph, *Sulfuricella* sp. Strain T08, Isolated from a Freshwater Lake

**DOI:** 10.1128/genomeA.00498-15

**Published:** 2015-05-21

**Authors:** Tomohiro Watanabe, Hisaya Kojima, Manabu Fukui

**Affiliations:** Institute of Low Temperature Science, Hokkaido University, Sapporo, Japan

## Abstract

*Sulfuricella* sp. strain T08 is a sulfur-oxidizing autotroph newly isolated from a freshwater lake in Japan. Strain T08 is the second isolate of the genus *Sulfuricella*. Here, we report the annotated draft genome sequence of the isolate.

## GENOME ANNOUNCEMENT

Betaproteobacteria have been recognized as major constituents of the sulfur-oxidizing bacterial community in freshwater lakes (1, 2). Recently, a new order, *Sulfuricellales*, was established in the class *Betaproteobacteria* to accommodate three freshwater sulfur oxidizers of two genera (3). The type genus of this order is the genus *Sulfuricella*, and members of this genus have been detected in various freshwater environments as nucleotide sequences. To date, *Sulfuricella denitrificans* skB26^T^ is the sole isolate belonging to the genus *Sulfuricella* (4), and its complete genome has been sequenced (5). In the present study, a novel member of the genus *Sulfuricella* was obtained, and its genome was sequenced.

*Sulfuricella* sp. strain T08 was isolated from a sample of large filamentous bacteria of the genus *Thioploca* obtained from sediment of a freshwater lake in Japan, Lake Okotanpe. The *Thioploca* sample was transferred into bicarbonate-buffered low-salt defined medium containing 10 mM S_2_O_3_ and 10 mM NaNO_3_ as the electron donor and acceptor, respectively (4). The headspace of the culture bottle was filled with N_2_/CO_2_ (80:20 [vol/vol]), and incubation was performed in the dark at 13°C. After several transfers, successive subcultures were performed in a slightly different medium, which has no NaCl and increased nitrate concentration (20 mM), at 20 to 22°C. Finally, a pure culture of strain T08 was obtained by agar shake dilution (6). The isolate grew autotrophically on thiosulfate and nitrate. During the growth, gas production was observed, and nitrite was not detected. Phylogenetic analysis of the 16S rRNA gene sequence revealed that strain T08 belongs to the genus *Sulfuricella*. The new isolate was deposited in the National Institute of Technology and Evaluation Biological Resource Center (NBRC) as NBRC 110684.

Genomic DNA was extracted using a cetyltrimethylammonium bromide method (7). For next-generation sequencing, a DNA library with 350-bp inserts was prepared using the TruSeq Nano DNA LT sample prep kit (Illumina). The library was sequenced using paired-end 100-bp reads on an Illumina HiSeq. A total of 10,283,118 high-quality filtered reads were assembled *de novo* using Velvet version 1.2.08, with a hash length of 95 bp. The resulting assembly comprised 78 scaffolds of 91 contigs totaling 3.4 Mb in size, with an average length of 43,892 bp. The scaffolds were annotated automatically using the Microbial Genome Annotation Pipeline (8). Functional annotation was performed using the KEGG Automatic Annotation Server (9). The wrongly predicted genes were manually inspected and corrected, as described previously (5). As a result, a total of 3,216 genes, 43 tRNAs, and 1 rRNA operon were predicted.

A BLASTp search revealed that strain T08 possesses a set of sulfur oxidation-related genes previously identified in the genome of *S. denitrificans* skB26^T^ (9). On the other hand, the amino acid sequences of 274 genes in the strain T08 genome share <70% coverage, 35% identity, and >1 × *e*^−5^ *E* value with the genes of strain skB26^T^. A detailed analysis of these genes will provide insight into phenotypic variation within the genus *Sulfuricella*, which is widespread in freshwater environments.

### Nucleotide sequence accession numbers. 

This whole-genome shotgun project has been deposited in DDBJ/EMBL/GenBank under the accession no. BBWF00000000. The version described in this paper is the first version, BBWF01000000.
